# ARE RECENTLY INCARCERATED OLDER ADULTS MORE LIKELY TO DEVELOP DEMENTIA THAN THE NEVER INCARCERATED?

**DOI:** 10.1093/geroni/igac059.525

**Published:** 2022-12-20

**Authors:** Amy Byers, Richard Fortinsky, Brie Williams, W John Boscardin, Yixia Li, Lisa Barry

**Affiliations:** University of California, San Francisco, San Francisco, California, United States; University of Connecticut Center on Aging, Farmington, Connecticut, United States; University of California, San Francisco, San Francisco, California, United States; University of California, San Francisco, San Francisco, California, United States; San Francisco VA Medical Center, San Francisco, California, United States; University of Connecticut Center on Aging, Farmington, Connecticut, United States

## Abstract

Increasing numbers of older adults are reentering the community following incarceration (i.e., reentry). Yet, their risk of developing dementia and mild cognitive impairment (MCI) is unknown. We leveraged a national cohort of veterans who experienced reentry at age ≥ 65 years (N=5,920) and compared their risk of dementia or MCI to an age- and sex-matched never incarcerated sample (N=29,600). Those in the reentry sample were incarcerated for ≤10 consecutive years, experienced reentry between 10/01/2012 and 9/30/2018, and did not have a pre-incarceration MCI or dementia diagnosis per VA and CMS healthcare records (N=5,920). Fine-Gray models, controlling for race and pre-incarceration chronic conditions, serious mental illness, traumatic brain injury, and posttraumatic stress disorder, derived hazard ratios (HRs) and 95% Confidence Intervals (CIs). Samples were 99% male with average age 70 (±4.3) years. Reentry adults had a higher proportion of non-Whites (29% vs. 17%; p< 0.001) and more chronic conditions (2.1[±1.8] vs. 1.6[±1.6] p< 0.001). MCI incidence did not differ between reentry and never incarcerated groups (3.1% vs. 2.6%; HR=1.03; 95%CI 0.86-1.24). However, risk of any dementia was higher in reentry older adults (7.2% vs. 5.0%; HR=1.27; 95%CI 1.12, 1.44), as was risk of specific subtypes, e.g., vascular dementia (1.9% vs. 1.2%; HR=1.37; 95% CI 1.06-1.75) and frontotemporal dementia (0.2% vs. 0.1%; HR=2.3; 95% CI 1.2, 4.3). Those reentering the community in late life following incarceration may be a group that is especially at risk of developing dementia. These findings raise awareness of the need for appropriate transition planning for this vulnerable group.

